# Supporting people with pain-related distress in primary care consultations: a qualitative study

**DOI:** 10.3399/BJGP.2022.0120

**Published:** 2022-08-09

**Authors:** Noureen A Shivji, Adam WA Geraghty, Hollie Birkinshaw, Tamar Pincus, Helen Johnson, Paul Little, Michael Moore, Beth Stuart, Carolyn A Chew-Graham

**Affiliations:** School of Medicine, Keele University, Keele.; Primary Care Research Centre, University of Southampton, Southampton.; Department of Psychology, University of London, London.; Department of Psychology, University of London, London.; School of Medicine, Keele University, Keele.; Primary Care Research Centre, University of Southampton, Southampton.; Primary Care Research Centre, University of Southampton, Southampton.; Primary Care Research Centre, University of Southampton, Southampton.; School of Medicine, Keele University, Keele; honorary professor of primary care mental health, Midlands Partnership NHS Foundation Trust, St George’s Hospital, Stafford.

**Keywords:** depression, general practitioners, pain, pain management, referral and consultation, self-management

## Abstract

**Background:**

Low mood and distress are commonly reported by people who have persistent musculoskeletal (MSK) pain, which may be labelled as ‘depression’. It is important to understand how pain-related distress is conceptualised and managed in primary care consultations.

**Aim:**

To explore understanding of pain-related distress and depression from the perspectives of people with persistent MSK pain and GPs.

**Design and setting:**

Qualitative study with people with persistent MSK pain and GPs from different parts of the UK.

**Method:**

Semi-structured interviews conducted remotely and data analysed thematically.

**Results:**

Most participants reported challenges in distinguishing between distress and depression in the context of persistent MSK pain, but also described strategies to make this distinction. Some people described how acceptance of their situation was key and involved optimism about the future and creation of a new identity. Some GPs expressed ‘therapeutic nihilism’, with uncertainty about the cause of pain and how to manage people with both persistent MSK pain and distress in primary care consultations, while GPs who could identify and build on optimism with patients described how to help the patient to move forwards.

**Conclusion:**

This study offers a framework for the primary care consultation with patients presenting with pain-related distress. GPs should recognise the impact of persistent MSK pain on the patient and support the person in coming to terms with their pain, explore how the person feels about the future, encourage optimism, and support self-management strategies.

## INTRODUCTION

Musculoskeletal (MSK) conditions account for 30.5% of all years lived with disability in the UK and can have a significant impact on wellbeing, with prevalence of depression estimated at three to four times greater than that of the general population.^1^^–^3 Distress and depression have been found to predict the transition to persistent pain.^4^^–^6

The majority of people with persistent pain and mental health symptoms are managed in primary care,^7^ and a central role for the GP is to respond to the patient’s concern and identify and manage a clinical disorder.^8^^,^^9^ GPs may find making the distinction between distress and depression difficult,^10^ and it is unclear how GPs work with and support people with pain and try to distinguish between pain-related distress and depression. The distinction between pain-related distress and depression is particularly important because of the conflicting messages received by primary care practitioners to better detect mental health problems (especially depression),^9^^,^^11^ while avoiding overmedicalising distress and thus overtreatment (especially with antidepressants).^12^^,^^13^

People living with persistent pain regard the psychological and social needs arising from their health problems to be at least as important as managing the pain itself.^14^ Patients may consider their emotional experience to be different to their perceived notions of ‘actual’ depression or mental illness.^15^

To add to this complexity, pain-related distress is qualitatively different from clinical depression,^16^ and current referral pathways and available interventions are suboptimal for people with persistent pain who are distressed.^17^ This may result in people with pain-related distress receiving unacceptable or inappropriate interventions.

The overall aims of this qualitative study were to explore how people with pain understand pain-related distress and how this is managed in primary care. The interviews explored the management offered for pain and distress, and interventions that people with pain have found to be helpful. It was aimed to explore perspectives and experiences of GPs managing and supporting people with persistent pain, how they distinguish between pain-related distress and depression, and to develop implications for successful outcomes to primary care consultations for people with pain-related distress.

## METHOD

The study employed a qualitative methodology with semi-structured interviews to explore the perspectives of people with persistent MSK pain and GPs in understanding and managing pain-related distress.

**Table:** How this fits in

Previous research has mainly focused on the impact of persistent pain on people’s lives, and the challenge of living with persistent pain. People with pain are often distressed, and this can be difficult for both patients and doctors to distinguish from depression. This study focuses on how GPs can work with and support people with pain, endeavour to distinguish between pain-related distress and depression, and achieve successful outcomes to the primary care consultation.

Patient and public involvement was integral to this study, with a patient advisory group (PAG) contributing to study design, public-facing documents, and analysis of findings. A GP stakeholder group contributed to the study design. Appropriate qualitative reporting guidelines are used.^18^

### Recruitment and participants

People with persistent pain were recruited using four methods: advertisements in public places (for example, pharmacies and shops), social media posts (Twitter and Facebook), local radio, and identification through general practice registers (searched by practice staff, supported by clinical research networks [CRNs]). This allowed the use of both convenience (recruitment using advertisements) and purposive sampling (when recruited through general practice). GPs were recruited using social media and professional networks (convenience sampling), ‘snowballing’,^19^ as well as through local CRNs in Wessex, Kent, Surrey, and Sussex using purposive sampling.

When potential participants (either people with pain or GPs) contacted the research team, eligibility was checked, and they were sent an information sheet and consent form. Once a completed consent form was received by the research team, the interview was arranged.

### Data generation

Interviews with all participants were conducted via telephone or using virtual software (such as Microsoft Teams), as preferred by participants. Informed consent was reconfirmed and recorded at the start of the interview. Interviews were conducted by a female health services researcher with a PhD and a background in nursing, and another female health services researcher with a PhD. Both are experienced researchers with qualitative methods expertise. They had no previous relationships with the interview participants, who were given participant information leaflets about the study before consenting to participate.

Demographic data (including age, sex, ethnicity, area lived in, employment, and educational attainment) were collected at the start of the interview for people with pain. GPs were asked to indicate age, sex, ethnicity, area worked in, number of sessions worked clinically, years of experience as a GP, and any area of expertise. These data were collected to contextualise the data and support description of the samples.

The interview topic guides were developed by the research team in collaboration with the study PAG and GP stakeholders. The topic guides were modified iteratively as data generation and analysis progressed and allowed exploration of perceived differences in pain-related distress and depression in people with persistent pain, and language used by participants to describe pain-related distress. The interview schedules were used flexibly to allow unanticipated topics to be explored and modified iteratively as data generation and analysis progressed. An example of the topic guides is presented in Supplementary Box S1.

All interviews were digitally audiorecorded and transcribed verbatim by a professional transcription company. Any identifiable information was removed from the transcripts by the researchers. Field notes were made during and immediately after each interview.

At the end of the interview, each participant was asked if they wished to receive a summary of the findings and/or publication(s) arising from the study. Participants were offered reimbursement for their time. A copy of the lay summary (based on initial analyses) was sent to those participants who requested it.

No further interviews were conducted with the participants. Transcripts were not sent to participants for their comments.

### Data analysis

Transcripts were uploaded into qualitative research software (NVivo, version 11) to aid data organisation. Thematic analysis was conducted by the whole research team, using the principles of constant comparison.^20^ Thematic analysis focuses on meaning across a dataset, allowing researchers to understand collective and shared experiences.^21^^,^^22^ The analysis was conducted iteratively, using an inductive approach to data analysis, where coding and theme development were guided by the content of the data obtained.^22^ One researcher initially coded and analysed all the transcripts, which were also analysed and coded by members of the entire research team involved in analysis, to ensure intercoder reliability.^21^^,^^22^ Having a research team with mixed professional backgrounds (including health service researchers, academic GPs, academic psychologists, and a patient co-investigator) allowed robust discussion on data interpretation and increased trustworthiness of analysis.^23^

Each dataset (people with pain and GPs) was analysed separately before themes across datasets were collated. A lay summary and analysis framework based on initial analysis were discussed at a PAG meeting and a preliminary stakeholder meeting with GPs, and their feedback was incorporated into further data generation and analysis. The research team continued with data collection and analysis until they were confident that no new meanings or themes could be identified from the transcripts, such that data saturation had been achieved.^24^

## RESULTS

### Demographics

In total, 21 interviews were conducted with people with persistent pain, and 21 more with GPs. The demographics of people with pain and GPs are presented in Tables 1 and 2. Mean duration of the interviews was around 50 minutes for both people with pain and GPs.

**Table 1.** Demographic characteristics of people with pain, *N* = 21

**Characteristic**	***N***	**%**
**Recruited via:**		
** Media**	12	57.1
** Snowballing**	2	9.5
** CRN**	7	33.3

**Sex**		
** Male**	7	33.3
** Female**	14	66.7

**Ethnicity**		
** White British**	17	81.0
** Asian British**	2	9.5
** African British**	2	9.5
** Other**	1	4.8

**Area of living**		
** Rural**	10	47.6
** Urban**	11	52.3

	**Mean**	**SD**

**Age, mean (SD)**	55.1	11.9

**Number of years living with pain (range 1–25 years)**	12.7	7.6

a *Media, including social media and adverts (online, print and radio). CRN = Clinical Research Network. SD = standard deviation.*

**Table 2.** Demographic characteristics of GPs, *N* = 21

**Characteristic**	***N***	**%**
**Recruited via:**		
** Media**	4	19.0
** Snowballing**	4	19.0
** PN**	8	38.1
** CRN**	5	23.8

**Sex**		
** Male**	13	61.9
** Female**	8	38.1

**Ethnicity**		
** White British**	15	71.4
** Asian British**	5	23.8
** Others**	1	4.8

	**Mean**	**SD**

**Age, mean (SD)**	47.3	10.4

**Clinical sessions per week**	4.5	2.2

**Experience in years**	17.6	11.0

a *Media, including social media and adverts (online, print and radio). CRN = Clinical Research Network. PN= Professional Network. SD = standard deviation.*

### Main themes

Three main themes were developed: ‘pain and distress are inter-linked’, ‘being stuck’, and ‘moving forwards’. An overarching thread of ‘recognising and dealing with uncertainty’ underpins all the themes.

Illustrative data are provided to support the analysis; data extracts are identified by participant number, self-disclosed sex, and age to provide contextual information.

### Distress and pain are interlinked

All participants reflected on how distress and pain are inseparable from each other. Distress was seen as a reaction to, and impact of, persistent pain.

#### Impact of pain

People with pain described the impact of pain on every aspect of their lives. They reported feeling overwhelmed by the pain, not being able to perform daily chores or pleasurable activities, and becoming dependent on others:

*‘Yes, because just some days, I just feel like I can’t even, you know do a load of laundry or cook dinner or, like simple things like that … ’*

(Participant [P]16, female [F], aged 35 years)

Persistent pain and its consequences were felt to have an impact on mood:

*‘If I have a bad day and all of it hurts, I can get quite upset. It does affect my mood. It irritates me that I can’t cut my toenails properly and when I’m in the shower. Like I say, it’s putting socks on and things like that. It just makes you so fed up.’*

(P12, F, aged 45 years)

This impact of pain on patients’ lives was recognised by GPs:

*‘They have very restricted lives quite often, they are very limited, they become very deconditioned, they are often having others in the family playing a caring role, even children. And there’s an overlap with fatigue and its quite severe emotional distress and they know we can’t make it better … ’*

(GP15, male [M], aged 60 years)

#### Uncertainty

The Invisibility of pain experienced was felt to add to distress and the fear of not being believed, and thus add to the impact of the pain on relationships, including with healthcare professionals:

*‘It’s so frustrating because you think are people just thinking I’m making it up. So, I suppose you know, the big aspect is I don’t do much at all.’*

(P10, F, aged 48 years)

*‘And that part is the hardest part, not just the fact you’re going through it, but that there’s nobody more willing to listen to you about it.’*

(P4, F, aged 39 years)

People with pain also expressed uncertainty about the future, whether the pain would continue, and if recovery would ever be possible:

*‘He* [GP, during consultation] *left me one long thought that as you get older it will get worse, so what I’ve been suffering, the increasing intensity and frequency over the last 20 years, will not stop, it’s going to carry on and* [yeah] *the frightening thing is I don’t know if I’ll get to the stage where I begin to lose control over what I do.’*

(P9, M, aged 71 years)

#### Pain, distress, and depression

Most participants described a cyclical and interactive relationship between pain and distress:

*‘There are people who unfortunately have a debilitating condition in which pain is an issue and if pain isn’t controlled then you go down this downward spiral of pain is not controlled, it makes your mood worse, which again will make your pain worse and then you kind of end up in a black hole where you’ve got two problems, pain, and a mood disorder … so it’s kind of like what came first, the chicken or the egg.’*

(GP5, F, aged 36 years)

Some GPs suggested that there was a linear relationship between distress and depression, offering a window of opportunity to intervene:

*‘So, you’ve got a pain, you’re fearful about what it is, it’s not going away so you’re worrying about the future and therefore that causes distress. And if that’s not addressed quick enough then it becomes depression because it’s not been resolved.’*

(GP10, F, aged 60 years)

Some people with pain recognised that they might have an underlying vulnerability to distress and depression:

*‘Well, I think that it’s taken some time to kind of work out, because I’ve always had poor mental health, I’ve had chronic pain since my mid to late twenties. The depression was already around before then, so you have to kind of unpick what is causing the depression or exacerbating the depression.’*

(P11, M, aged 55 years)

GPs described how symptoms owing to pain, distress, and depression overlapped, making it challenging for them to differentiate depression and distress:

*‘I don’t think there is a clear distinction. I think it’s a spectrum. So, distress merges into depression. There’s not a hard and fast distinction.’*

(GP20, M, aged 65 years)

*‘Often, it’s more of a blurred situation and there may be elements of both … I think it’s just something as a practitioner you need to be aware of whenever you’re having these consultations so you make sure you can at least explore that as a possibility. I don’t have a way of distinguishing specifically the two.’*

(GP2, M, aged 46 years)

These uncertainties are then played out in the primary care consultation.

### Being stuck

People with pain described ‘getting stuck’, seeing no way forward:

*‘You don’t see any way forward, there’s no solution to the problem so obviously it’s a mental reaction, you know, you are not in control because the pain is overriding everything so they* [people with pain] *want the pain to go away and they can’t find a way of it going away, so they become distressed and depressed with it as well.’*

(P5, M, aged 62 years)

While some GPs conceptualised pain-related distress as a medical disorder needing treatment, others suggested that distress was a normal response to pain. Many GPs, however, expressed ideas of ‘therapeutic nihilism’, with limited options available to them to manage patients with persistent pain and distress:

*‘I think the problem* [is] *there isn’t a lot that we can do really, so apart from making sure that you know people have had all the tests they’ve meant to have had, for things that we can fix, making sure they’ve been referred to the pain clinic, and that we’re trying appropriate medications, thinking about their mental health, there isn’t a lot that we can do. And I think you know in that way we kind of quite similar maybe to the patients, this condition as well, there’s not a lot they can do to make things better*. *’*

(GP13, M, aged 41 years)

GPs recognised that patients could be dissatisfied with consultations, which in turn led to the GP feeling frustrated:

*‘And I guess with if we’re talking about pain or chronic pain … they want their pain to go away and that’s not always possible with chronic pain at the moment, let’s say. And so, we end up kind of having this conversation that doesn’t go anywhere because both parties are probably sort of quite dissatisfied of where, there’s not much progress actually made. And what happens essentially is they probably will go to another GP, for example, try their luck and the same story starts again. So yes, this is quite frustrating, I’d say at times.’*

(GP8, M, aged 44 years)

Some people with pain reported limited expectations of what could be done to help them manage their pain and deal with their distress:

*‘*. *.. I think the doctors have sort of given me everything they can. Chronic pain* [clinic] *has said they’ve done as much as they can. So, I’ve gone through all their programmes and stuff like that, so it’s just a case of they just check up on my meds and things like that. So, there’s nothing much else.’*

(P10, F, aged 48 years)

### Moving forwards

#### Being believed

Patients contrasted experiences of being dismissed by GPs with more positive consultations, which included feeling that they were listened to and believed by a GP whom they trusted:

*‘I felt brushed off by my GP, he didn’t have any other solutions other than giving me drugs which he knows I don’t like*. *’*

(P17, F, aged 74 years)

*‘Well, my GP is good she does really understand, she knows how, she’s been with me for the last few years, so she understands. But I can understand … you can’t do miracles, I understand that* … *’*

(P20, F, aged 59 years)

GPs understood patients’ desires to be heard and believed, and illustrated how listening could be used as one of the strategies to manage patients with persistent pain:

*‘I think kind of explaining to the patient that you can see the impact it’s having on them and that you know you believe them, that they’ve got this pain and it’s severe and it’s not getting better, I think that helps, at least you care … ’*

(GP13, M, aged 41 years)

The importance of being treated empathetically as a person was highlighted, along with discussing and sharing uncertainties:

*‘If you speak to people and they’re sympathetic to you, it is a help … you know but I appreciate that not everyone would have that.’*

(P5, M, aged 62 years)

GPs described how they needed to work with the patient to unpick these uncertainties and deal with their own to move on:

*‘I think obviously when we talk to the patient if they just say that, if they’re fixated just on the pain and the body so that’s a primary symptom, then I find that more a sort of distress with the chronic pain. If they tell me that there’s other symptoms you know they’ve got poor appetite, poor energy, poor concentration, some of the symptoms, that will open the door for doing, you know looking at the PHQ-9 and assessment for depression. Just really how they present … and I’m further questioning what other symptoms they allude to.’*

(GP21, M, aged 46 years)

#### Regaining control

Coming to terms with or accepting pain was seen as important by people with lived experience of pain:

*‘... the way that I try and become more positive is just to carry on and get on with whatever it is. I suppose it makes me feel like I still accomplished whatever it was I was going to accomplish.’*

(P12, F, aged 45 years)

GPs emphasised the importance of encouraging patients to accept the pain and subsequent distress to move forwards:

*‘What most people would recognise as an acute stress reaction to something, because most of us, you know, something happens, yeah, we get upset about it, we get stressed about it or whatever, acutely, and then we kind of stabilise after a while and I wouldn’t say we get used to it but you kind of … I don’t know what term I should use, but you kind of just accept and then you move on, and it doesn’t become so intrusive.’*

(GP5, F, aged 36 years)

People with pain described the need to be optimistic to deal with their current situation:

*‘You know when you spiral down a little bit then you start thinking of all the negative reasons of everything. If you looked at the positive stuff, then perhaps you wouldn’t be in that frame of mind.’*

(P21, M aged 54 years)

GPs highlighted the significance of fostering ‘optimism’ within patients living with persistent pain and distress to open possibilities for people with pain to adapt to their current life situation, rather than focusing on what has been lost:

*‘I try to be positive because I want them to be exercising and I want them to be doing what they can to help this rather than accepting this as some form of negative, debilitating problem that they’ve just got to take loads of pain relief.’*

(GP2, M, aged 46 years)

People with pain described working on constructing a new identity, which included accepting their pain, and giving a sense of adapting to and restoring a new life:

*‘I still have, what do they call it when you have pain, the triggers, you know it* [pain course] *taught me to avoid the triggers, it taught me to deal with the triggers so when you get a really, really bad phase that you go through on how to deal with all of that psychologically and physically. So, I just have to live with it* [pain] *and do what I was taught to do, you know, and I thank goodness that I went on that course.’*

(P19, F, aged 66 years)

#### Agreeing solutions

GPs emphasised the importance of having an established relationship with the patient:

*‘I would say that’s the first and the most important thing and having continuity of care with the same doctor or the same clinician and not seeing a different person every time. It should be someone who really gets to know the patient and understands their point of view and the patient then, over time, gets to know and trust that doctor. I don’t think you can do it in one consultation.’*

(GP19, M, aged 48 years)

People with pain stressed the need to work with their GP to develop and agree a management plan to deal with both pain and distress:

*‘It’s only because I spoke to my GP recently and she was just like saying how was I and stuff and I was just talking about stuff and she said, “Right, you really need to be recommended to the mental health team, are you happy with that?” And I was like, “Well yeah, I don’t mind”.’*

(P10, F, aged 48 years)

Key to moving forwards was trying to distinguish between distress and depression and plan management accordingly. Some GPs described how identifying a cause for low mood might help them to distinguish between distress and depression:

*‘Distress is when I think you know there’s something that’s happened specifically and the distress or the emotional upset is related directly to that thing. Depression is more when it becomes more generalised, you feel you are not winning with every aspect of your life and everything is … and there may not be a specific reason for it and it’s prolonged, you know, it goes on beyond what most people would recognise as an acute stress reaction to something.’*

(GP5, F, aged 36 years)

Other GPs described how they based their management decisions on whether there was a previous history of depression:

*‘… I don’t know whether they feel helpless and hopeless and that leads to clinical depression, or they have got a pre-existing low mood that makes their pain threshold low.’*

(GP6, M, aged 38 years)

Some GPs said they considered severity of symptoms to be central to distinguishing between distress and depression in the context of pain. It was common for these GPs to mention the use of severity scales such as the Patient Health Questionnaire-9 (PHQ-9) to help them make that distinction.^25^

*‘So that kind of usual like screening questions for depression really. And then if they said, oh, like, the answer to those questions was yes, then you can assess more and there are some scoring questionnaires we can use for depression if we want to, like the PHQ-9 … ’*

(GP13, M, aged 41 years)

Once this distinction was made, GPs described how they were more certain in offering support and negotiating a management plan:

*‘I also use treatment as a way of differentiating whether something is becoming … is more of a longstanding problem or not, so if people are responding to the treatment and becoming more active, less distressed on the second occasion, that’s more of an indication to me that this is something that can be treated. When people come back and it hasn’t made a difference and they’re saying that you know they haven’t managed to change how they think or how they behave, then that’s more of a trigger for me to be thinking oh this is something more of a depression.’*

(GP7, M, aged 60 years)

**Figure 1.**
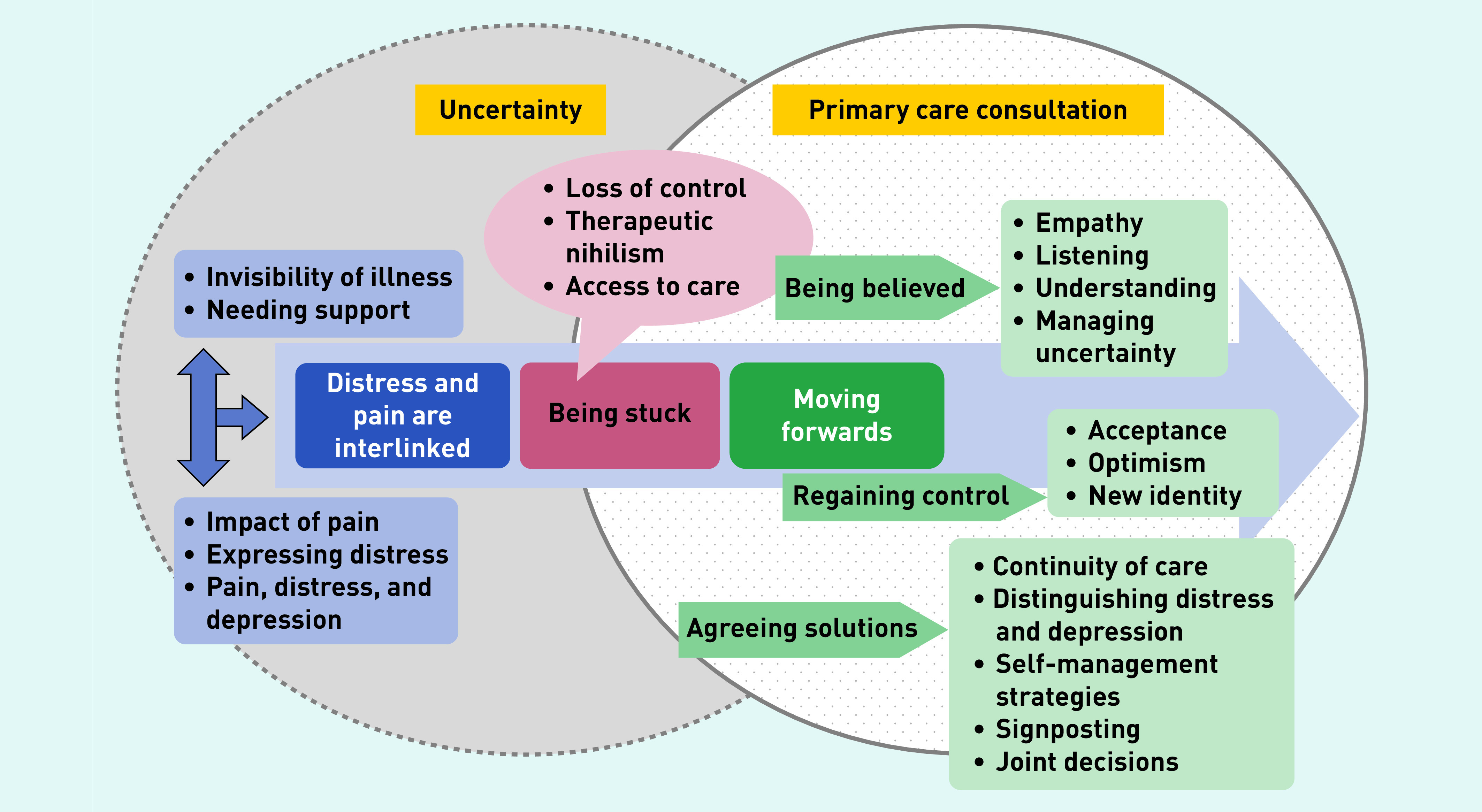
*Framework to support patients with persistent pain within the primary care consultation*

The findings were discussed with the PAG and drawn together in Figure 1, which represents a framework to support the primary care consultation.

## DISCUSSION

### Summary

This study offers a framework (Figure 1) for the primary care consultation for patients presenting with pain-related distress. This framework incorporates hearing the patient’s story,^26^ recognising and empathising with the impact of pain on the patient, supporting the person in coming to terms with their pain, exploring how the person feels about the future, and encouraging optimism and engagement with self-management strategies. The key to this is recognising and managing their own and their patient’s uncertainty about the cause of pain, and attempting to distinguish between distress and depression.

### Strengths and limitations

This study presents comparative accounts of people with pain and GPs, and has allowed the development of a framework, with input from people with lived experience, which could help clinicians manage patients with persistent pain in the primary care consultation. Strengths of this study include multiple methods of recruitment of both people with persistent MSK pain and GPs across England. The research team is multidisciplinary and includes a person with lived experience of pain, who contributed to all aspects of the study.^24^ The study design, data analysis, and framework were discussed with the PAG.

There was limited ethnic diversity within the sample of people with pain, and half had a degree. Recruitment using social media may have restricted participation to the study to people who are digitally literate; however, parallel recruitment using CRNs will have reduced the impact of this limitation. Nearly half of GPs interviewed expressed an interest in the management of MSK problems.

### Comparison with existing literature

It has been established that GPs find it challenging to distinguish between emotional distress and depression,^10^ and that patients recruited from primary care considered their emotional experience to be different to their perceived notions of ‘actual’ depression or mental illness.^15^ Patients’ understanding of their pain and associated distress often reflect the complexity of their lives and may not fit neatly into biomedical models.^15^ It is suggested that before acceptable management can be negotiated, there is a need here to develop models of psychological symptoms that draw on patient experience and help them understand the nature of their experience.^15^ This complexity is exaggerated when distress is associated with pain, and it is this complexity that is brought to the primary care consultation.

The clinical encounter between the doctor and the patient has both tremendous practical and ideological importance for the discipline of primary care. In general practice, however, biomedical reductionism is impossible in the way that more general critiques of medical knowledge and practice suggest,^27^ and the importance of patient-centred care was recognised.^28^ In addition, all primary care consultations entail some kind of uncertainty, which patients and GPs need to negotiate and manage^29^ to achieve a satisfactory outcome to the interaction.

### Implications for practice

This study offers a framework that GPs can draw on to support them in consultations with people with persistent pain. This work is timely, as there has been much criticism of the recent National Institute for Health and Care Excellence (NICE) guideline for assessment of chronic pain and management of chronic primary pain.^30^

While the guideline highlights the importance of patient-centred care and shared decision making, little advice about how to do this is included, and the reduction in management options has been highlighted with the suggestion that it leaves GPs impotent.^31^ Indeed, the Faculty of Pain Medicine (Royal College of Anaesthetists) outline the risks associated with this guideline,^32^^,^^33^ including reduction of medication options and decommissioning of pain clinics. The challenge is that these recommendations and possible consequences come at a time of unprecedented workload in primary care.^34^

This guideline does, however, stress that patients should be assessed meaningfully to develop a more constructive shared understanding of how their pain experience is shaped. Patients need to be given time to tell their stories and be partners in their care. Time spent with a GP they know, who listens to and believes them, will avoid frustration on both sides and enable more productive consultations. Of key importance is that both the GP and person with pain identify distress and distinguish this from a depressive illness. Fostering optimism and looking forwards can be achieved in the context of a trusting GP–patient relationship.

The NICE recommendations play to the strengths of primary care practice, and it is hoped that this framework, developed with a PAG, will support GPs in managing this group of patients, leading to improved satisfaction for both GPs and patients.

## References

[1] Vos T, Flaxman AD, Naghavi M (2012). Years lived with disability (YLDs) for 1160 sequelae of 289 diseases and injuries 1990–2010a systematic analysis for the Global Burden of Disease Study 2010. Lancet.

[2] Sullivan MJL, Reesor K, Mikail S, Fisher R (1992). The treatment of depression in chronic low back pain: review and recommendations. Pain.

[3] Tunks ER, Crook J, Weir R (2008). Epidemiology of chronic pain with psychological comorbidity: prevalence, risk, course, and prognosis. Can J Psychiatry.

[4] Chou R, Shekelle P (2010). Will this patient develop persistent disabling low back pain?. JAMA.

[5] Mallen CD, Peat G, Thomas E (2007). Prognostic factors for musculoskeletal pain in primary care: a systematic review. Br J Gen Pract.

[6] Pincus T, Burton AK, Vogel S, Field AP (2002). A systematic review of psychological factors as predictors of chronicity/disability in prospective cohorts of low back pain. Spine (Phila Pa 1976).

[7] Ferenchick EK, Ramanuj P, Pincus HA (2019). Depression in primary care: part 1 — screening and diagnosis. BMJ.

[8] Bower P, Gilbody S (2005). Managing common mental health disorders in primary care: conceptual models and evidence base. BMJ.

[9] Mitchell AJ, Vaze A, Rao S (2009). Clinical diagnosis of depression in primary care: a meta-analysis. Lancet.

[10] Geraghty AW, Santer M, Beavis C (2019). ‘I mean what is depression?’ A qualitative exploration of UK GPs’ perceptions of distinctions between emotional distress and depressive disorder. BMJ Open.

[11] Nuyen J, Volkers AC, Verhaak PF (2005). Accuracy of diagnosing depression in primary care: the impact of chronic somatic and psychiatric comorbidity. Psychol Med.

[12] Dowrick C, Frances A (2013). Medicalising unhappiness: new classification of depression risks more patients being put on drug treatment from which they will not benefit. BMJ.

[13] Elliott AM, Smith BH, Penny KI (1999). The epidemiology of chronic pain in the community. Lancet.

[14] Froud R, Patterson S, Eldridge S (2014). A systematic review and meta-synthesis of the impact of low back pain on people’s lives. BMC Musculoskelet Disord.

[15] Geraghty AW, Santer M, Williams S (2017). ‘You feel like your whole world is caving in’: a qualitative study of primary care patients’ conceptualisations of emotional distress. Health (London).

[16] Nuyen J, Volkers AC, Verhaak PFM (2005). Accuracy of diagnosing depression in primary care: the impact of chronic somatic and psychiatric co-morbidity. Psychol Med.

[17] Rusu AC, Pincus T, Morley S (2012). Depressed pain patients differ from other depressed groups: examination of cognitive content in a sentence completion task. Pain.

[18] Tong A, Sainsbury P, Craig J (2007). Consolidated criteria for reporting qualitative research (COREQ): a 32-item checklist for interviews and focus groups. Int J Qual Health Care.

[19] Atkinson R, Flint J (2001). Accessing hidden and hard-to-reach populations: snowball research strategies. Social Res Update.

[20] Guest G, MacQueen KM, Namey EE (2012). Applied thematic analysis.

[21] Braun V, Clarke V (2006). Using thematic analysis in psychology. Qual Res Psychol.

[22] Braun V, Clarke V, Cooper H, Camic PM, Long DL (2012). Thematic analysis. APA handbook of research methods in psychology, 2: research designs: quantitative, qualitative, neuropsychological, and biological.

[23] Henwood K, Pidgeon N (1992). Qualitative research and psychological theorizing. Br J Psychol.

[24] Saunders B, Sim J, Kingstone T (2018). Saturation in qualitative research: exploring its conceptualization and operationalization. Qual Quant.

[25] Kroenke K PHQ-9 (Patient Health Questionnaire-9). Objectifies degree of depression severity. https://www.mdcalc.com/calc/1725/phq-9-patient-health-questionnaire-9.

[26] Launer J (2022). Is taking a history outmoded? Why doctors should listen to stories instead. Postgrad Med J.

[27] May CM, Allison G, Chapple A (2004). Framing the doctor–patient relationship in chronic illness: a comparative study of general practitioners’ accounts. Sociol Health Illn.

[28] May C, Mead N, Dowrick C, Frith L (1999). Patient-centredness: a history. General practice and ethics: uncertainty and responsibility.

[29] Lian OS, Nettleton S, Wifstad Å, Dowrick C (2021). Negotiating uncertainty in clinical encounters: a narrative exploration of naturally occurring primary care consultations. Soc Sci Med.

[30] National Institute for Health and Care Excellence (2021). Chronic pain (primary and secondary) in over 16s: assessment of all chronic pain and management of chronic primary pain NG193.

[31] Smith BH, Colvin LA, Donaldson-Bruce A, Birt A (2021). Drugs for chronic pain: we still need them. Br J Gen Pract.

[32] Faculty of Pain Medicine of the Royal College of Anaesthetists (2021). FPM concerns regarding new NICE Chronic Pain Guidelines. https://fpm.ac.uk/fpm-concerns-regarding-new-nice-chronic-pain-guidelines.

[33] Eccleston C, Aldington D, Moore A, de C Williams AC (2021). Pragmatic but flawed: the NICE guideline on chronic pain. Lancet.

[34] Gerada C (2021). General practice in crisis: stop skinning the cat. Br J Gen Pract.

